# Fingolimod in a patient with heart failure on the background of pulmonary arterial hypertension and coronary artery disease

**DOI:** 10.1186/1471-2377-14-126

**Published:** 2014-06-07

**Authors:** Katja Thomas, Hagen Schrötter, Michael Halank, Tjalf Ziemssen

**Affiliations:** 1 Center of Clinical Neuroscience, Department of Neurology, Neurological University Clinic Dresden, University Clinic Carl Gustav Carus Dresden, University of Technology Dresden, Fetscherstr, 74, 01307 Dresden, Germany; 2 Dresden Heart Center, University Clinic Carl Gustav Carus Dresden, University of Technology Dresden, Fetscherstr, 76, 01307 Dresden, Germany; 3 Department of Internal Medicine I, University Clinic Carl Gustav Carus Dresden, University of Technology Dresden, Fetscherstr, 74, 01307 Dresden, Germany

**Keywords:** Multiple sclerosis, Fingolimod, Pulmonary arterial hypertension

## Abstract

**Background:**

Fingolimod is the first oral immunomodulatory therapy approved for highly active relapsing remitting multiple sclerosis. Based on the distribution pattern of fingolimod interacting sphingosine-1-phosphat receptors in organism including immune system and cardiovascular system clinical monitoring of patients and evaluation of adverse events are recommended. Despite extensive data on cardiovascular safety, experience with fingolimod in patients with concomitant cardiological disease, especially within the pulmonary circulation, is rare.

**Case presentation:**

We report the case of a 46-year-old woman presented with relapsing remitting multiple sclerosis and severe idiopathic pulmonary arterial hypertension. Fingolimod was initiated because of disease activity of multiple sclerosis with two relapses and gadolinium-enhancing lesions in MRI. The patient demonstrated stable disease course of idiopathic pulmonary arterial hypertension when fingolimod was started. Fingolimod therapy did not alter or even worsen the pulmonary or cardiovascular conditions during first dose application as well as follow up of nine months.

**Conclusion:**

In this report, we present the first case of fingolimod treatment in a patient with highly active multiple sclerosis and severe idiopathic pulmonary arterial hypertension. We suggest an interdisciplinary approach with detailed cardiopulmonary monitoring for safety in such patients.

## Background

Fingolimod is approved as second line disease modifying drug for highly active relapsing remitting multiple sclerosis (MS) patients. In its phosphorylated form it serves as sphingosine-1-phosphat (S1P) receptor analogue, orchestrating relevant mechanisms in the immune system but also cardiovascular system [1]. There are only few reports on S1P-triggered effects on cardiovascular function within systemic or pulmonary circulation.

## Case presentation

The 46-year-old female patient was diagnosed with relapsing-remitting MS in 2005. She started on interferon-beta 1a in 2005 presenting a stable disease course in the following years. In 2010, severe idiopathic pulmonary arterial hypertension (IPAH) and one-vessel coronary artery disease were diagnosed after progressive exertional dyspnea, dizziness, marked restricted physical performance and mild stenocardia since 10 months, characterizing heart failure stage III of classification of the New York Heart Association (NYHA). Echocardiography showed right ventricular hypertrophy, moderate reduction of left ventricular and severe reduction of right ventricular function, paradox septum deviation and severe tricuspid valve insufficiency with an estimated systolic pressure of 90 mmHg (Table 1). Routine echocardiography 12 months before was without abnormal results. Combined left and right heart catheterization demonstrated one-vessel coronary artery disease and severe pre-capillary pulmonary hypertension with a mean pulmonary arterial pressure of 66 mmHg (normal: 10–20 mmHg), left ventricular end-diastolic pressure of 12 mmHg (normal: 3–12 mmHg), cardiac index of 1.5 L/min/m^2^ (normal: 2.5-4.0 L/min/m^2^) and pulmonary vascular resistance of 14.1 wood units (normal: 0.25-1.6 wood units). The initial level of N-terminal pro-hormone of brain natriuretic peptide (NT-proBNP) presented with an increase up to 207 pmol/L (normal: ≤17 pmol/L). One week after placing a drug eluding stent in the left main coronary artery (LMCA) control echocardiography revealed an unchanged severe pulmonary hypertension with near normalized left ventricular function. Diagnosis of severe IPAH was established by exclusion of other causes of pulmonary hypertension according to the diagnostic criteria reported by Galie et al. [2]. Compression of the LMCA by an enlarged pulmonary artery was excluded by CT-angiography of the pulmonary arteries. The patient started on the endothelin receptor antagonist ambrisentan and phosphodiesterase inhibitor tadalafil. By this treatment, the patient reported an improvement of physical performance, no more dizziness and stenocardia as well as a stable disease course corresponding to heart failure of stage NYHA II. In further evaluations, mild right ventricular hypertrophy and insufficiency, mild tricuspid valve insufficiency, decrease of right ventricular-systolic pressure in echocardiography, normal lung function, mild impairment in diffusion capacity of carbon monoxide and mild hypocapnia was detectable in the following years (Table 1).

**Table 1:** Right and left ventricular parameter before and after fingolimod application

	**Diagnosed PAH**	**Pre-FTY**	**Post-FTY 1 month**	**3 month follow up**	**9 month follow up**
NYHA	III	II	II	II	II
HR [bpm]	80	77	65	60	68
BP [mmHg]	120/88	130/80	110/90	122/85	125/85
RVESP [mmHg]	90	70	65	n.d.	55
TAPSE [mm]	13	21	n.d.	n.d.	20
Right ventricle hypertrophy	mild	mild	mild	n.d.	mild
LV EF	45%	60%	60%	n.d.	60%
NT-proBNP [pmol/L]	207	17	16	15	11
pH (cap.)	7.46	7.54	7.52	7.47	7.43
PO2 (cap.) [kPa]	12.3	10.1	11.7	10.4	10.4
PCO2 (cap.) [kPa]	3.9	4.6	3.5	4.6	4,72
Respiratory resistance	Normal	Normal	Normal	Normal	Normal
Obstructive ventilation	Normal	Normal	Normal	Normal	Normal
Restrictive ventilation	Normal	Normal	Normal	Normal	Normal
Diffusion respiration	TLCO SB 49%	TLCO SB 51%	TLCO SB 53%	TLCO SB 50%	TLCO SB 49%

FTY, Fingolimod; n.d., not done. Reference values: RVESP: 15–30 mmHg; TAPSE: < 20 mm; LV-EF, left ventricular ejection fraction: 50–70%; NT-proBNP: ≤ 17 pmol/L, pH (capillary): 7.35-7.45; PO2 (capillary), partial pressure of oxygen: 11.1-14.4 kPa; PCO2 (capillary), partial pressure of carbon dioxide: 4.27-6.4 kPa; TLCO SB, transfer coefficient of lung for carbon monoxide: > 60%.

In 2013, she demonstrated with significant clinical disease activity of MS with two relapses and MRI activity with three gadolinium-enhancing lesions and an Expanded Disability Status Scale (EDSS) of 4.0. Because of positive JC-virus serology, fingolimod was selected as second line treatment. Before first dose application, extensive cardio-pulmonary testing demonstrated stable cardiovascular parameters as described above (Table 1). The baseline electrocardiogram (ECG) showed a sinus rhythm with a right bundle branch block. During first dose, heart rate (HR) decreased at maximum by 13 bpm at 4 hours and reached baseline HR 6 hours after initial fingolimod administration. 12 lead ECG as well as Holter ECG did not demonstrate any significant abnormalities (Table 2). Blood pressure (BP) in lying and tilted up position presented within normal range during the whole 6 hours monitoring (Table 2). The patient did not complain any findings in the first 6 hours after fingolimod application. Cardio-pulmonary testing one week, one, three and nine months after first dose application demonstrated stable parameters in ECG, BP, echocardiography, body plethysmography and blood gas analysis (Table 1). Especially relevant parameters of right ventricular and pulmonary circulation including right ventricular hypertrophy, right ventricular end-systolic pressure (RVESP), right-ventricular ejection fraction and tricuspid annular plane systolic excursion (TAPSE) kept stable on fingolimod treatment (Table 1). Detailed testing of the autonomic nervous system demonstrated no change of the efferent sympathetic regulation and left ventricular function after orthostatic stress before, 2 hours, 4 hours, and 6 hours after fingolimod first dose application as well as in three and nine month follow up (Table 2). Maximal heart rate variability measured by expiration/inspiration quotient (E/I) during deep metronomic breathing showed a decrease to 1.06, which stabilized over time back to 1.09 (Table 2) [3]. There were no additional cardiac or pulmonary symptoms than known before fingolimod treatment.

**Table 2:** Parameter of systemic circulation during passive orthostatic tilt test before and after fingolimod application

	**Pre-FTY baseline**			**2 h post-FTY**			**4 h post-FTY**			**6 h post-FTY**			**3 month follow-up**			**9 month follow-up**		
	→	↑	→	→	↑	→	→	↑	→	→	↑	→	→	↑	→	→	↑	→
HR [bpm]	74	84	80	70	73	66	61	64	59	74	76	74	65	70	65	60	64	62
Psyst [mmHg]	143	130	156	123	108	129	134	114	133	127	118	134	156	126	131	149	127	136
Pdiast [mmHg]	85	82	92	65	65	72	71	67	72	68	70	75	74	72	71	77	75	77
TPR [mmHg *s/ml]	1.43	1.55	1.5	0.68	0.76	0.83	0.92	0.86	0.9	0.69	0.88	0.85	0.81	1.06	0.93	1.1	1.41	1.23
CO [lpm]	3.9	3.5	4.3	8.4	5.9	6.9	6.7	6.3	6.5	7.9	6.1	7.1	7.4	4.9	6.2	5.9	4.5	5.1
SV |ml]	50	43	54	119	83	67	106	97	116	109	78	103	116	72	96	102	70	83
E/I		1.16			1.13			1.14			1.16			1.06			1.09	

FTY, Fingolimod. → lying position; ↑ tilted position. Reference values: HR: 60–100 bpm; Psyst, systolic blood pressure: 100–140 mmHg; Pdiast, diastolic blood pressure: 60–90 mmHg; TPR, total peripheral resistance: 0.6-1.3 mmHg*s/ml; CO, cardiac output: 4–8 lpm; SV, stroke volume: 55–100 ml; E/I: > 1.11.

## Conclusion

In this report, we present the first case of fingolimod treatment in a patient with severe IPAH and coronary heart disease. Although there were no short- and long-term side effects by fingolimod in this patient, patients with cardiovascular disease should be carefully selected for fingolimod therapy. It is necessary to evaluate existing cardiovascular conditions inclusive the medication critically. An interdisciplinary approach with frequent cardio-pulmonary exams like ECG, BP, echocardiography, body plethysmography and blood gas analysis should be complemented to monitor and ensure safety in such patients.

Fingolimod is known as S1P receptor analogue that mediates inhibition of lymphocyte egress from lymph nodes into the peripheral blood [1]. Beside the immune system S1P receptors are also relevant for further signallining inside the human body including the cardiovascular and pulmonary system. Based on the natural distribution pattern of S1P receptors especially on artrial myocytes, fingolimod can cause transient bradycardia or decrease of atrioventricular conduction [4]. It is known, that S1P promotes contraction of bronchi and especially bronchial smooth muscle cells [5,6]. Furthermore increased risk of arterial hypertension and vascular dysfunction caused by S1P receptor subtypes in endothelium and smooth muscle cell layer is discussed during fingolimod treatment [7,8]. Today, there is extensive experience regarding safety and tolerability during first dose application and long-term treatment with fingolimod in MS patients [9]. Several fingolimod-related cardiovascular side effects have been discussed so far including prolonged symptomatic bradycardia, AV block or non-fatal cardiac asystole at first dose application or ventricular arrhythmia after five months of treatment [10-12]. A critical arterial vasospasm of the left arm seven days after initiation of fingolimod was also presented [13]. In a patient with Wolff-Parkinson-White syndrome, no cardiovascular interaction with fingolimod was described [14]. Nevertheless, data on fingolimod treated patients with definite concomitant cardiac diseases are rare, especially if the right side of the heart is involved.

IPAH is characterized by elevated pulmonary arterial pressure and pulmonary vascular resistance causing right ventricular hypertrophy and insufficiency. Early intervention is necessary to avoid right heart failure and sudden death. In our case, symptoms and objective assessment associated with IPAH improved after treatment initiation. Fingolimod was started during a stabilized disease course of IPAH over two years. Fingolimod therapy did not alter or even worsen the pulmonary or cardiovascular conditions during first dose application as well as during follow up.

### Consent

Written informed consent was obtained from the patient for publication of this Case report and any accompanying images. A copy of the written consent is available for review by the Editor of this journal.

## Competing interests

KT received personal compensation for speaking from Novartis and Bayer and received travel support to scientific meetings from Biogen idec, Sanofi, Teva and Genzyme. HS declares that he has no competing of interest. MH received fees for speaking at conferences and/or consultations from Actelion, AOP, Bayer, GSK, Lilly, Novartis and Pfizer and received travel and accommodation support to scientific meetings from Actelion, Bayer, GSK, Lilly, Novartis and Pfizer.TZ received personal compensation from Biogen Idec, Bayer, Novartis, Sanofi, Teva, Synthon for consulting services. Additionally he received financial support for research activities from Bayer, Biogen Idec, Novartis, Teva, Sanofi Aventis.

## Authros’ contributions

KT, HS, MH and TZ diagnosed and treated the patient. KT wrote the draft manuscript, prepared and analysed testings and data. HS and MH were involved drafting the manuscript. TZ initiated the study, reviewed and edited the manuscript and approved the final version. All authors read and approved the final manuscript.

## Pre-publication history

The pre-publication history for this paper can be accessed here:

http://www.biomedcentral.com/1471-2377/14/126/prepub
